# *De novo* chromosome level assembly of a plant genome from long read sequence data

**DOI:** 10.1111/tpj.15583

**Published:** 2021-12-02

**Authors:** Priyanka Sharma, Ardashir Kharabian Masouleh, Bruce Topp, Agnelo Furtado, Robert J. Henry

**Affiliations:** ^1^ Queensland Alliance for Agriculture and Food Innovation University of Queensland Brisbane QLD 4072 Australia; ^2^ ARC Centre of Excellence for Plant Success in Nature and Agriculture University of Queensland Brisbane QLD 4072 Australia

**Keywords:** *de novo* genome assembly, HiFiasm, HiFi reads, nuclear genome, plastid genome, mitochondrial genome, nuclear ribosomal RNA, technical advance, *Macadamia jansenii*

## Abstract

Recent advances in the sequencing and assembly of plant genomes have allowed the generation of genomes with increasing contiguity and sequence accuracy. Chromosome level genome assemblies using sequence contigs generated from long read sequencing have involved the use of proximity analysis (Hi‐C) or traditional genetic maps to guide the placement of sequence contigs within chromosomes. The development of highly accurate long reads by repeated sequencing of circularized DNA (HiFi; PacBio) has greatly increased the size of contigs. We now report the use of HiFiasm to assemble the genome of *Macadamia jansenii*, a genome that has been used as a model to test sequencing and assembly. This achieved almost complete chromosome level assembly from the sequence data alone without the need for higher level chromosome map information. Eight of the 14 chromosomes were represented by a single large contig (six with telomere repeats at both ends) and the other six assembled from two to four main contigs. The small number of chromosome breaks appears to be the result of highly repetitive regions including ribosomal genes that cannot be assembled by these approaches. *De novo* assembly of near complete chromosome level plant genomes now appears possible using these sequencing and assembly tools. Further targeted strategies might allow these remaining gaps to be closed.

## Significance Statement

*De novo* assembly of a near compete chromosome level plant genome is now possible using long read sequencing and assembly tools.

## INTRODUCTION

Reference genome sequences are a key resource for plant science. The challenge of producing a complete genome sequence has been greatly reduced by advances in both DNA sequencing (Hon et al., 2020; Levy and Myers, 2016) and sequence assembly tools (Chen et al., 2017; Phillippy, 2017). Final assembly of chromosome level genomes has relied upon evidence other than the sequence data alone, such as genetic maps (Fierst, 2015; Yu et al., 2019).

Advancements in the field of sequencing, assembly and scaffolding technologies, along with the rapid increase in the amount of freely available genomic data (https://www.ncbi.nlm.nih.gov/genbank/statistics), has greatly facilitated the development of highly accurate *de novo* assemblers.

Short‐read *de novo* assemblers are not efficient in assembling the complex and long repetitive regions of plant genomes, such as centromeres and telomeres (Liao et al., 2019). To address this limitation, long read sequencing technologies, also known as third generation sequencers, have been developed. However, these long reads from PacBio (Menlo Park, CA, USA) and Oxford Nanopore (Oxford, UK) have been less accurate, with an average base calling accuracy of 90% compared to the 99.9% accuracy of the Illumina (San Diego, CA, USA) reads (Amarasinghe et al., 2020; Shendure et al., 2017). Hybrid assembly pipelines have often been used to assemble many genomes, aiming to overcome the shortcomings of both the long reads and short reads. This has allowed assembly of larger contigs from complex genomes. However, to achieve chromosome level genome assembly, scaffolding of the contigs was usually required. Analysis of sequence proximity in the chromatin by methods such as Hi‐C has made this possible (Dudchenko et al., 2017; Kaplan and Dekker, 2013).

Recent advances in long read sequencing technology have allowed a single molecule to be sequenced multiple times to produce long high fidelity reads (HiFi; PacBio) with a base level accuracy of 99.9% (Wenger et al., 2019). We have used *Macadamia jansenii* to compare methods for the sequencing and assembly of plant genomes (Murigneux et al., 2020; Sharma et al., 2021a). This genome has a size (approximately 800 Mb) typical of many plant genomes but with a relatively low heterozygosity (Sharma et al., 2021b). Assembly of this genome using highly accurate circular consensus sequencing (CCS) reads (HiFi; PacBio) using the HiFiasm assembly tool (Cheng et al., 2021) was found to give a more contiguous genome than that obtained with earlier longer continuous long reads (CLR; PacBio) (Sharma et al., 2021a). The HiFiasm assembler has been used to successfully assemble genomes of *Fragaria* × *ananassa* (garden strawberry), *Rana muscosa* (mountain yellow‐legged frog) and *Sequoia sempervirens* (California redwood) (Cheng et al., 2021). Recently, HiFiasm was reported to allow highly contiguous assembly of plant genomes (Driguez et al., 2021). We now report the near complete chromosome level assembly of the *M. jansenii* genome from HiFi reads with the HiFiasm assembly tool, as well as an analysis of the assembled genome against a Hi‐C chromosome level assembly.

## RESULTS

### HiFiasm assembly

The estimated genome size of the *M. jansenii* genome is 780 Mb (Murigneux et al., 2020). The size of the primary Hifiasm assembly was 826 Mb, including 779 contigs (Table S1), with the longest contig of 71.9 Mb and an average contig length of 1 Mb. busco analysis (https://busco.ezlab.org) showed that the assembly covered 99.6% of universal single copy genes (Table 1). The contigs generated in this assembly were characterized in three groups based upon their size: large contigs (>1 Mb); medium size contigs (between 1 Mb and 100 kb) and small contigs (<100 kb).

**Table 1:** HiFiasm contigs in different size categories and comparison of primary and haploid assemblies generated from HiFiasm genome assembler tool

	Number of contigs	Assembly length (Mb)	N50 (Mb)	N75 (Mb)	busco (%)
HiFiasm assembly					
Total contigs	779	826	46	25	99.6
Contigs >40 Mb	10	524	50	46	68.7
Contigs >10 Mb	19	746	48	39	93.9
Contigs >1 Mb	30	784	46	30	99.1
Contigs >100 kb	94	805	46	27	99.0
Between 100 kb and 1 Mb	64	20	0.49	0.22	0.20
Between 10 kb and 100 kb	685	22	0.032	0.028	0.00
Comparison of HiFiasm primary and haploid assemblies					
Primary assembly	779	827	46.1	25	99.60
Hap 1_assembly	879	816	24.4	8.9	98.80
Hap 2_assembly	363	776	14.3	5.4	97.90
Hap 1 >1 Mb	96	736	16.4	6.8	96.70
Hap 2 >1 Mb	72	766	24.5	12.3	98.10

#### Larger size contigs >1 Mb

There were 30 contigs greater than 1 Mb in length. These contigs alone provided a good assembly with an N50 of 46 Mb and a busco score of 99.1% (Table 1). Dotplot analysis against the Hi‐C assembly (Sharma et al., 2021b) showed that, of the nine contigs more than 46 Mb in length, eight correspond to complete Hi‐C pseudomolecules (i.e. each contig corresponds to a single chromosome; chromosomes 1, 4, 5, 6, 10, 11, 13 and 14) (Figure 2a). One contig (Ptg000010|), corresponded to a large part of the second largest chromosome (chromosome 2) and another two contigs of approximately 25 and 2.7 Mb covered the other parts of this chromosome (Figure 2b and Tables 2 and 3). The 14 contigs between 4 and 46 Mb in size covered the remaining six chromosomes, in combinations of two to four contigs. Five of the contigs between 1 and 4 Mb in size corresponded to nuclear ribosomal RNA sequences, and the other two contigs matched parts of chromosome 2 and 7 (Figure 2b and Tables 2 and 3).

**Table 2:** Chromosomal location of HiFiasm contigs >1 Mb

Contig id >1 Mb	Length in bp	Hi‐C pseudo‐molecule corresponding HiFiasm contigs
ptg000016l	71 935 981	Chr 1 + Ribo RNA
ptg000003l	57 251 071	Chr 6
ptg000017l	57 081 251	Chr 4
ptg000011l	56 513 637	Chr 5
ptg000004l	49 863 231	Chr 10
ptg000012l	48 320 516	Chr 11
ptg000023l	47 997 562	Chr 13
ptg000010l	46 138 073	Chr 2 + Ribo RNA
ptg000008l	46 131 124	Chr 14
ptg000014l	43 049 961	Chr 9
ptg000009l	39 279 660	Chr 3
ptg000002l	29 700 554	Chr 8
ptg000001l	26 771 894	Chr 12
ptg000006l	25 189 511	Chr 2
ptg000007l	23 138 637	Chr 7 + Ribo RNA
ptg000013l	22 539 440	Chr 8
ptg000020l	22 399 594	Chr 7
ptg000052l	20 335 125	Chr 12
ptg000021l	13 354 688	Chr 3
ptg000019l	8 098 418	Chr 7
ptg000022l	6 676 624	Chr 3
ptg000005l	6 127 021	Chr 9
ptg000072l	4 271 045	Chr 12
ptg000018l	2 743 534	Ribo RNA
ptg000025l	2 713 795	Part of Chr 2
ptg000062l	1 651 603	Ribo RNA
ptg000074l	1 299 006	Ribo RNA
ptg000034l	1 171 806	Ribo RNA
ptg000036l	1 154 310	Ribo RNA
ptg000033l	1 122 141	Part of Chr 7

**Table 3:** HiFiasm contigs ( 1 Mb) covering each of the Hi‐C pseudo‐molecules

*Macadamia jansenii* HiC pseudo‐molecules (A)	Size of HiC pseudo‐molecules (B)	HiFiasm contigs corresponding to HiC scaffolds (C)	HiFiasm contigs length (with explanation) (D)	HiFiasm combined contigs length (E)	Extra HiFiasm length (HiFiasm contig length − HiC scaffold length) (E − B)
Chr 1	67 682 215	ptg000016l	71 93 5981	71 935 981	4 253 766
Chr 2	63 669 590	ptg000006I + ptg000025I + ptg000010I	74 041 379 (=25 189 511 + 2 713 795 + 46 138 073)	74 041 379	10 371 789
Chr 3	58 143 993	ptg000021I + ptg000009l + ptg000022I	59 310 972 **(**=13 354 688 + 39 279 660 + 6 676 624)	59 310 972	1 166 979
Chr 4	56 076 407	ptg000017l	57 081 251	57 081 251	1 004 844
Chr 5	5 522 0784	ptg000011l	56 513 637	56 513 637	1 292 853
Chr 6	53 595 462	ptg000003l	57 251 071	57 251 071	3 655 609
Chr 7	52 077 970	ptg000019I + ptg000020I +ptg000033l + ptg000007I	54 758 790 (=8 098 418 + 22 399 594 + 1 122 141 + 23 138 637)	54 758 790	2 680 820
Chr 8	49 563 658	ptg000013I + ptg000002l	5 223 9994 (=22 539 440 + 29 700 554)	52 239 994	2 676 336
Chr 9	49 085 581	ptg000014l + ptg000005	4 917 6982 (=43 049 961 + 6 127 021)	49 176 982	91 401
Chr 10	48 974 653	ptg000004l	4 986 3231	49 863 231	888 578
Chr 11	47 698 009	ptg000012l	4 832 0516	48 320 516	622 507
Chr 12	46 713 600	ptg000001l + ptg000072I + ptg000052I	51 378 064 (=26 771 894 + 4 271 045 + 20 335 125)	51 378 064	4 664 464
Chr 13	45 610 911	ptg000023l	47 997 562	47 997 562	23 86 651
Chr 14	45 288 529	ptg000008l	46 131 124	46 131 124	842 595

#### Medium size contigs

**Figure 1:**
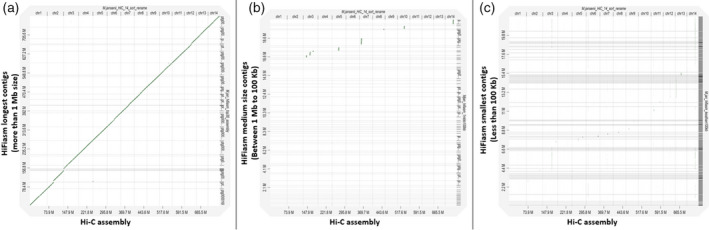
Dotplot of *Macadamia jansenii* Hi‐C genome assembly against HiFiasm contigs. (a) HiFiasm longest contigs (>1 Mb size), (b) HiFiasm medium size contigs (<1 Mb and >100 kb) and (c) HiFiasm smallest contigs (<100 kb).

There were 64 contigs between 1 Mb and 100 kb in size. These contigs had 0% busco genes (Table 1). Only eight contigs in the range between 100 and 824 kb corresponded to seven Hi‐C pseudo‐molecules (with an alignment block length of more than 100 kb) (Figure 1b; Figures S2 and Figure S3; Tables S2 and S3). Out of these eight contigs, five corresponded to the terminal part of the Hi‐C pseudo‐molecules and three corresponded to the non‐terminal regions of Hi‐C chromosomes 3 and 7, marked as red starts in Figure S2(a,b). Most of the medium size contigs corresponded to ribosomal RNA genes (Figure 5b) and one contig of 183 kb corresponded to a chloroplast assembly (Figure 3b). None of the contigs showed similarity with mitochondrial sequences (Figure 4b).

#### Smaller contigs

There were 685 contigs between 10 and 100 kb in size. Most of these small size contigs from the HiFiasm assembly corresponded to small portions of the chloroplast and mitochondrial genomes. These contigs aligned together covered the complete organelle genomes (Figures 3c and 4c). However, a few of contigs corresponded to nuclear ribosomal RNA sequences (Figure 5c). This contig set also showed 0% busco genes (Table 1).

### Influence of data volume

HiFiasm assembly from CCS reads from two individual single molecule, real time (SMRT) sequencing cells and the combined data is given in Table S1. A HiFiasm assembly generated from the 10× CCS data produced 4511 contigs with an assembly of 909 Mb and N50 of 0.38 Mb, whereas a larger CCS file with 18× coverage generated an assembly with less contigs (1058), a shorter assembly length (833 Mb) and an improved N50 of 4.4 Mb (Table S1). The 18× assembly was closer to the combined CCS assembly (and the Hi‐C assembly) than the 10× assembly.

Haploid assembly details are given in Table 1. The haploid 1 assembly had a greater number of contigs than the haploid 2 assembly. The busco results were similar for the two haploid and primary assemblies as all assemblies were relatively complete.

### Comparison with Hi‐C assembly

**Figure 2:**
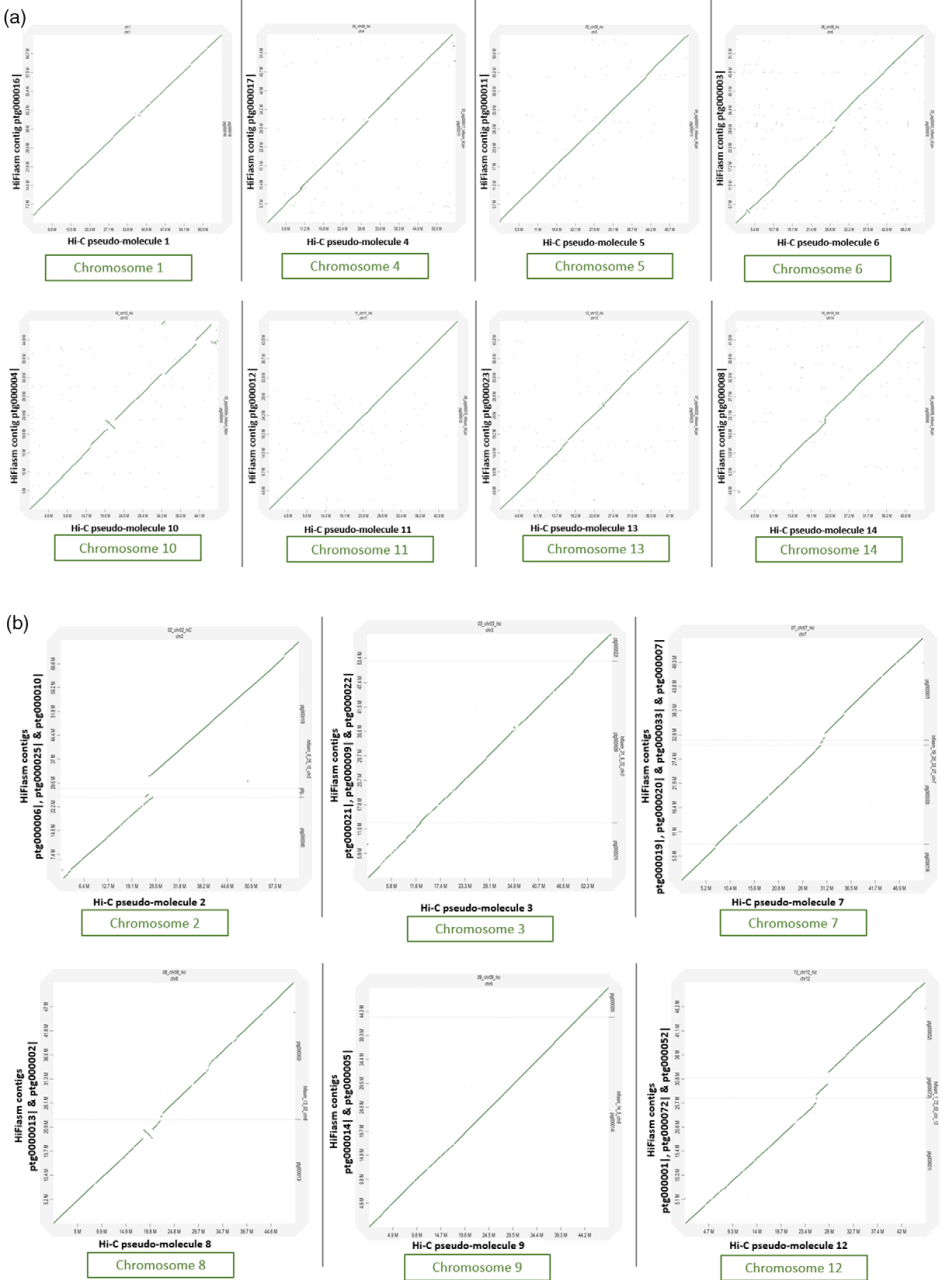
Dotplots of HiFiasm contigs against Hi‐C pseudo‐molecules. (a) Pseudo‐molecules that are covered by a single HiFiasm contig. (b) Pseudo‐molecules that are covered by more than one HiFiasm contig.

A dotplot analysis of 14 pseudo‐molecules of *M. jansenii* Hi‐C assembly against the HiFiasm assembly is shown in Figure 1. The dotplot of contigs >1 Mb in size showed a complete match of 25 contigs (out of total 30) with the 14 Hi‐C pseudo‐molecules (Sharma et al., 2021b) (Figure 1a). The remaining five large contigs did not contribute to the genome assembly. They were composed of nuclear ribosomal RNA sequences. Chromosomes 1, 4, 5, 6, 10, 11, 13 and 14 were covered by a single contig of the HiFiasm assembly (Figure 2a), two chromosomes (Chr 8 and 9) were covered by two contigs, chromosomes 2, 3 and 12 were covered by three contigs, and chromosome 7 was covered by four contigs (Figure 2b, Tables 2 and 3).

Analysis of the sequence at the ends of the HiFiasm contigs (Table 4) showed that the eight Hi‐C pseudo‐molecules (1, 4, 5, 6, 10, 11, 13 and 14) covered by single HiFiasm contigs had telomere repeats at both ends, except for pseudo‐molecules 1 and 5, which had a telomere at one end and an 18s ribosomal RNA on the other terminal. The other two pseudo‐molecules that were covered by two contigs (Chr 8 and Chr 9) had telomere sequences at one end of each contig. Chromosomes 2, 3 and 12 were covered by three contigs. In the case of chromosome 12, two contigs had telomere repeats at one end indicating their position at the end of the chromosome. One had 5S RNA gene sequences at the other end, confirming the match with 5S RNA sequences on the end of the middle contig. Chromosome 3 (covered by three contigs) also had two contigs with telomere repeats, confirming their terminal position in the chromosome. Similarly, chromosome 7 (covered by four contigs) had telomere repeats at one end of two contigs, indicating their position at the end of the chromosome and another two in the middle of the chromosome.

**Table 4:** Presence of telomere repeats and rRNA at the ends of HiFiasm contigs

Hi‐C pseudo‐molecule	HiFiasm contigs	Terminal 1 (HiFiasm contig)	Terminal 2 (HiFiasm contig)
Hi‐C pseudo‐molecules covered by a single HiFiasm contig			
Chr 1	ptg000016l	Telomere	18S rRNA
Chr 4	ptg000017l	Telomere	Telomere
Chr 5	ptg000011l	Telomere	18S rRNA
Chr 6	ptg000003l	Telomere	Telomere
Chr 10	ptg000004l	Telomere	Telomere
Chr 11	ptg000012l	Telomere	Telomere
Chr 13	ptg000023l	Telomere	Telomere
Chr 14	ptg000008l	Telomere	Telomere
Hi‐C pseudo‐molecules covered by more than one HiFiasm contig			
Chr 2	ptg000006I	–	Telomere
	ptg000025I	–	28S rRNA
	ptg000010I	18S rRNA	28S rRNA
Chr 3	ptg000021I	–	Telomere
	ptg000009I	–	–
	ptg000022I	–	Telomere
Chr 7	ptg000019I	–	Telomere
	ptg000020I	–	–
	ptg000033I	–	–
	ptg000007I	Telomere	–
Chr 8	ptg000013I	Telomere	Repeats
	ptg000002l	Telomere	Repeats
Chr 9	ptg000014l	Telomere	–
	ptg000005	Telomere	–
Chr 12	ptg000001l	Telomere	–
	ptg000072I	–	5S rRNA
	ptg000052I	Telomere	5S rRNA

### Organelle genome analysis

**Figure 3:**
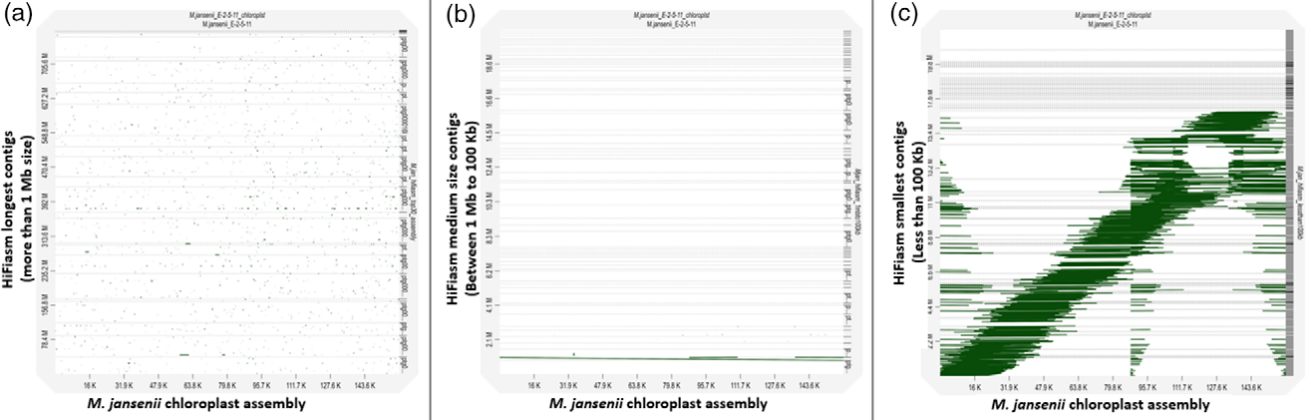
Dotplot of *Macadamia jansenii* chloroplast genome sequence against HiFiasm contigs. (a) HiFiasm longest contigs (>1 Mb size), (b) HiFiasm medium size contigs (<1 Mb and >100 kb) and (c) HiFiasm smallest contigs (<100 kb).

Dotplot analysis of a 159 Mb full length chloroplast genome assembled using the GetOrganelle toolkit (Jin et al., 2020) against the HiFiasm genome assembly indicated the insertion of small fragments of chloroplast sequences in the nuclear genome assembly (Figure 3a; Figure S1A), which also align with previously reported Hi‐C assembly results (Sharma et al., 2021b) (Figure S1B). Among the middle size contig set, only one contig (ptg0000186|) of 183 Mb aligned with the chloroplast genome (Figure 3b). Contig ptg000186| covered the complete chloroplast genome including the two inverted repeat regions of the chloroplast (Figure S4). Another HiFiasm middle size contig, ptg000066|, also showed some similarity with the chloroplast assembly and also aligned with the terminal end of Hi‐C chromosome 14 (Figure S5). Analysis of the smaller size contigs showed that the majority of these contigs contained some fragments of the chloroplast assembly (Figure 3c).

**Figure 4:**
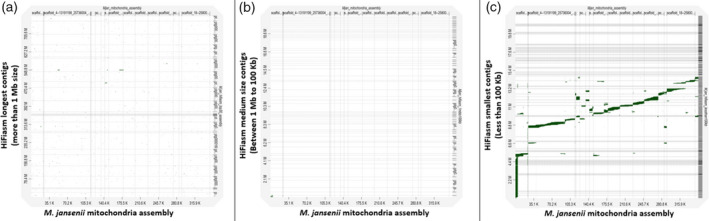
Dotplot of *Macadamia jansenii* mitochondria genome sequence against three sets of HiFiasm contigs. (a) HiFiasm longest contigs (>1 Mb size), (b) HiFiasm medium size contigs (<1 Mb and >100 kb) and (c) HiFiasm smallest contigs (<100 kb).

Mitochondrial sequence analysis revealed that the size of the *de novo* mitochondrial assembly was 351 kb. Analysis against the HiFiasm assembly indicated the presence of mitochondrial sequences in the smallest set of contigs. The majority of these contigs cover small fragments of the mitochondria genome (Figure 4c), whereas, in the larger contig set (>1 Mb), only a few contigs showed some similarity with mitochondrial sequences. These represent the mitochondria sequences inserted in the nuclear genome (Figure 4a), which aligns with the dotplot result of Hi‐C assembly (Figure S1B[b]). The middle size contigs did not show the presence of any mitochondria sequences in the dotplot analysis (Figure 4b).

### Nuclear ribosomal RNA gene sequences analysis

**Figure 5:**
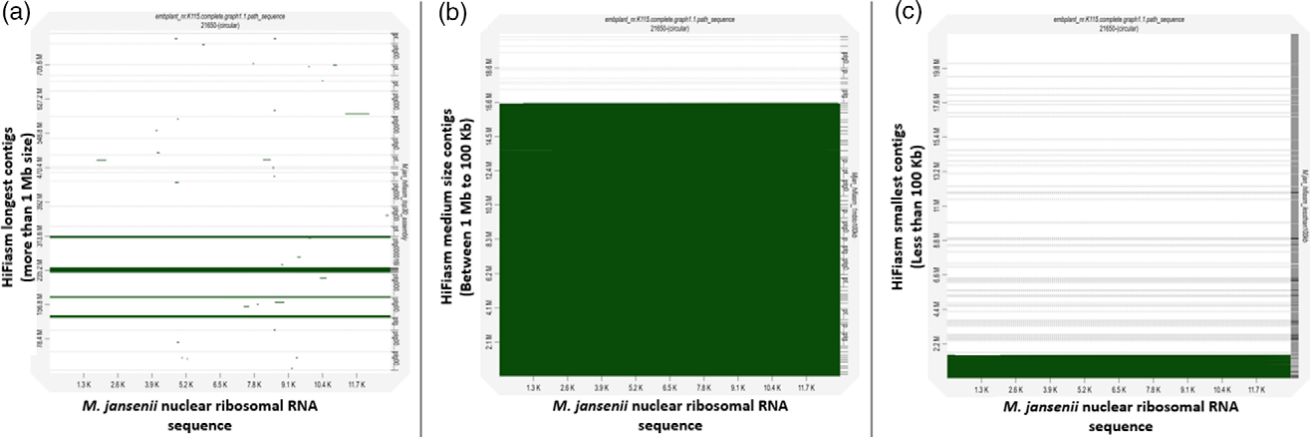
Dotplot of *Macadamia jansenii* nuclear ribosomal RNA sequence against HiFiasm contigs. (a) HiFiasm longest contigs (>1 Mb size), (b) HiFiasm medium size contigs (<1 Mb and >100 kb) and (c) HiFiasm smallest contigs (<100 kb).

Dotplot analysis of nuclear ribosomal RNA sequences showed matches with the majority of the middle size contigs, with a small number of contigs from the smaller set of contigs having ribosomal RNA sequences (Figure 5b,c).

### Analysis of repeat elements

The HiFiasm contigs were longer than the corresponding Hi‐C pseudomolecules (Table 3). This is probably because the HiFiasm contigs included a larger proportion of repetitive elements than the corresponding Hi‐C pseudomolecules (Table 5). The longer chromosome had a generally higher content of repetitive elements, suggesting that the presence of these repeat regions explained their greater size. The HiFiasm assemblies included more repetitive elements in the larger chromosomes but lower repeat content in the smaller chromosomes, largely as a result of the inclusion of less unclassified repeats in the HiFiasm assemblies of the smaller chromosomes.

**Table 5:** Comparative repetitive elements of Hi‐C pseudo‐molecules and HiFiasm assembly

*Macadamia jansenii* pseudo‐molecules	Genome assembler	Size of pseudo‐molecules	Total repeats (%)	LINE (%)	LTR (%)	DNA elements (%)	Unclassified (%)	Simple repeats (%)
Chr 1	Hi‐C	67 682 215	62	4.13	30.3	0.52	26.8	0.64
	HiFiasm	71 935 981	62.2	3.88	34.8	0.58	22.6	0.65
Chr 2	Hi‐C	63 669 590	66	3.31	38.2	1.12	23.3	0.86
	HiFiasm	74 041 379	68	2.98	39.6	1.22	23.8	0.44
Chr 3	Hi‐C	58 143 993	52.3	6.15	20.5	1.13	24.2	0.67
	HiFiasm	59 310 972	54.3	7.93	20.7	0.97	24.1	0.72
Chr 4	Hi‐C	56 076 407	55.1	6.26	22.8	0.79	24.3	1.13
	HiFiasm	57 081 251	57.2	7.21	21.6	0.90	26.56	1.23
Chr 5	Hi‐C	55 220 784	53.1	3.27	31.4	0.96	17.0	0.78
	HiFiasm	56 513 637	54.3	3.47	31.6	0.75	17.9	0.85
Chr 6	Hi‐C	53 595 462	55.1	8.75	19.9	1.18	25.5	0.79
	HiFiasm	57 251 071	58.8	9.47	22.6	1.43	24.0	1.44
Chr 7	Hi‐C	52 077 970	52.9	7.04	21.3	1.42	22.4	0.85
	HiFiasm	54 758 790	51.2	6.65	21.3	1.25	21.4	0.79
Chr 8	Hi‐C	49 563 658	44.0	5.39	15.5	0.64	21.3	1.25
	HiFiasm	52 239 994	41.8	6.25	22.0	1.30	12.3	0
Chr 9	Hi‐C	49 085 581	48.1	5.39	18.4	1.76	22.0	0.86
	HiFiasm	49 176 982	45.9	5.41	24.8	1.65	14.0	0
Chr 10	Hi‐C	48 974 653	48.1	6.24	17.3	0.90	22.8	1.02
	HiFiasm	49 863 231	44.7	5.91	22.9	2.82	13.1	0
Chr 11	Hi‐C	47 698 009	48.1	6.24	17.3	0.90	22.8	1.02
	HiFiasm	48 320 516	44.7	4.01	26.3	2.82	11.6	0
Chr 12	Hi‐C	46 713 600	44.6	4.47	16.5	1.39	21.3	0.85
	HiFiasm	51 378 064	25.6	4.47	21.2	2.44	11.7	0
Chr 13	Hi‐C	45 610 911	42.2	5.52	13.7	0.70	21.1	1.31
	HiFiasm	47 997 562	39.7	5.42	18.8	1.63	13.8	0
Chr 14	Hi‐C	45 288 529	42.5	5.82	12.9	0.96	21.5	1.50
	HiFiasm	46 131 124	41.1	5.53	19.6	1.94	13.9	0

## DISCUSSION

This era of genomics is continuing to advance with improved sequencing technologies and the potential to sequence all recorded species on earth (Lewin et al., 2018). Accurate chromosome level genome assembly requires accurate reads, high genome coverage and long read length. This has typically involved the use of very high coverage and data from multiple sequencing platforms along with mapping of Hi‐C technologies to achieve chromosome level assemblies. However, the combination of high sequence accuracy in a long read in HiFi reads (99.8% accuracy at around 15 kb average length) provides the option to assemble a complete genome using a single sequencing technology (Cheng et al., 2021) and with a more readily obtainable genome coverage (Wenger et al., 2019).

In the present study, we have combined the benefit of the highly accurate reads with an improved assembly tool HiFiasm (Cheng et al., 2021). HiFi read genome coverage of 28–40×, for plant genomes within the range of 700−1000 Mb size, was sufficient to generate high quality assemblies with Mb contig sizes (Sharma et al., 2021b). The DNA extracted from *M. jansenii* may have contained some impurities that reduced the efficiency of the DNA sequencing. Two SMRT cells were required to generate 28× genome coverage with CCS reads. For some samples, this may be possible with one single run providing the required coverage if sufficient DNA purity is achieved, reducing the cost of obtaining sufficient sequence. When the two individual CCS runs of 10× and 18× were assembled separately using HiFiasm, the final assembly was very fragmented (N50 of 0.38 and 4.4 Mb, respectively) for *M. jansenii* (Table S1), whereas the combined 28× gave a highly contiguous assembly with N50 of 46.1 Mb and 99.6% BUSCO results. The combined CCS run results suggests that, if the isolation method resulted in high purity DNA, a single run with less coverage may be sufficient to assemble the genome. The higher base‐calling accuracy by HiFi improves the assembly accuracy by bypassing many time‐consuming and heavy computational requirement steps in the assembly workflow. The *M. jansenii* assembly from HiFiasm using HiFi sequencing data produced a near chromosome level assembly, with eight contigs covering eight complete Hi‐C pseudo‐molecules and another six chromosomes, being covered by only one to four breaks and a total of 17 contigs. Chromosomes 1 and 5 had 18S ribosomal RNA genes at one end, suggesting that these repeats near the end of the chromosome had prevented assembly to the telomere. Chromosome 12 was interrupted by 5S ribosomal RNA genes. For a plant with approximately 800 Mb of data, we estimate a high‐quality chromosome level assembly could be produced within 1 week from the plant material, if the DNA extraction step is well established.

This highly contiguous *M. jansenii* chromosome level assembly will help achieve a better understanding of the genome of macadamias. All four species of *Macadamia* are listed as threatened under Australian legislation (Mast et al., 2008), although *M. jansenii* is particularly endangered because of its very low population size (<200 plants in the wild) (Shapcott and Powell, 2011). The highly accurate genome assembly will facilitate its conservation and use in breeding. *Macadamia jansenii* has small inedible nuts (Gross and Weston, 1992); however, as a result of its small tree size and narrow root spread, it is being tested as a rootstock and in hybrids with the commercial species *Macadamia intergrifolia* (Alam et al., 2018). The HiFiasm assembly (busco 99%) is much better than the Hi‐C assembly (busco 97%) (Sharma et al., 2021b), suggesting the incorporation of some regions missing in the Hi‐C assembly.

The initiative to complete the genome assembly of almost all living organisms (Koepfli et al., 2015; Lewin et al., 2018) requires a highly efficient assembly method with sustainable financial, computational and time requirements without compromising on genome accuracy. Contiguity and completeness should be taken into consideration (Rhie et al., 2021). Our analysis suggests that HiFiasm assembly with the HiFi reads may require almost no further scaffolding for the plants with similar genome size of approximately 800 Mb. Analysis of the nature of the few remaining regions of the genome that are not assembled in these analyses may allow the development of targeted strategies to complete these assemblies. Analysis of the sequences at the ends of the contigs formed by HiFiasm assembly of HiFi reads may identify those contigs that have been interrupted by repetitive sequences that cannot be assembled *de novo*. This technology is successfully assembling regions with high levels of the repeat sequences that make up more than 50% of the *M. jansenii* genome (Sharma et al., 2021b). It may be that the very high accuracy of the HiFi reads detects minor variations in repeat sequences that allow their unique assembly and that only perfect repeats that are longer than the HiFi reads create a barrier to assembly. The present study suggests that more than half of the total chromosomes could be assembled telomere to telomere for the plants with a genome size of approximately 800 Mb, whereas plants with larger genome sizes may require some additional methods for complete assembly. Assemblies of larger genomes have been shown to require a higher level of coverage with long read data to achieve the same size of assembled contigs (Sharma et al., 2021a). The chromosomes covered by more than one contig have some end sequences that indicate how they should be connected to other contigs. The present study also suggests that the large ribosomal gene clusters in the genome of plants may be one of the few limitations to complete assembly. This would suggest that sequence analysis of the ends of contigs could be used to guide high level assembly of the genome. However, additional information may be required for plants with very large and complex genomes. This approach will be useful for producing plant genomes generating high quality *de novo* chromosome level assemblies, especially for laboratories with limited financial, technical and computational resources.

## METHODS

### Sequencing data

Short‐read (Illumina) sequencing data were from Murigneux et al. (2020) and long read data (PacBio HiFi) were from Sharma et al. (2021a).

### HiFiasm assembly

The HiFiasm genome assembly (Cheng et al., 2021) was generated using the High Performance Computing facility at the University of Queensland. For assembly, 24 core processing units and 120 Gb of memory was employed. Default settings of the HiFiasm assembler were used to assemble heterozygous genomes with built‐in duplication purging parameters. The HiFiasm output directory consists of two haploid (1 and 2), one primary contig and one alternate haplotig GFA graph files. Each halplotig and one primary contig GFA file was converted to FASTA format using the awk command.

### Analysis of assembly

The primary HiFiasm assembly of *M. jansenii* included 779 contigs that were categorised into three subsets: (i) contigs <1 Mb size; (ii) contigs <1 Mb and more than 100 kb size; and (iii) contigs <100 kb size. Along with the main primary and two haploid assemblies, all three sets of primary contig subsets were passed through analysis using quast (Gurevich et al., 2013), busco (Simão et al., 2015) and repeatmodeler (Humann et al., 2019). The telomere sequences in the HiFiasm contigs were identified using the bioserf platform (https://bioserf.org) (Somanathan and Baysdorfer, 2018). Ribosomal RNA and other protein coding genes at the terminal end of the HiFiasm contigs were identified using an ncbi blast search (https://blast.ncbi.nlm.nih.gov). Ribosomal RNA in the contigs was identified using Barrnap (https://github.com/tseemann/barrnap) (Seemann, 2013) with default settings for eukaryotes.

### Comparison with Hi‐C assembly

The HiFiasm contigs were compared with the *M. jansenii* 14 pseudo‐molecules from the Hi‐C assembly (Sharma et al., 2021b) using the online interactive D‐Genies dotplot tool (Cabanettes and Klopp, 2018) to compare two genomes using Minimap2 and, for alignments, dotplot images were created after selecting the ‘sort contigs’ option, selecting the ‘minimum identity’ parameter at 0.75 and checking the ‘strong precision’ tick box.

### Characterisation of organelle genomes content of HiFiasm contigs

A reference mitochondrial genome, chloroplast genome and nuclear ribosomal RNA sequence from this sample were assembled from Illumina raw reads (Murigneux et al., 2020) using the GetOrganelle toolkit (Jin et al., 2020) with default parameters. The HiFiasm contigs (779) were compared with the organellar and ribosomal sequences in dotplots.

## AUTHOR CONTRIBUTIONS

RJH, AF, AKM and BT designed the study and supervised the project. PS and AKM were responsible for genome assembly and analysis. PS, AF, AKM and RJH were responsible for data analysis. PS, RJH and AF were responsible for the tables and figures. PS and RJH drafted the manuscript. PS was responsible for data deposition. All authors edited and approved the final manuscript submitted for publication.

## CONFLICT OF INTEREST

The authors declare no conflict of interest.

## Supporting information


**Figure S1**. (A) Dotplot of *Macadamia jansenii* Hifiasm longest contigs (more than 1 Mb) against the (a) chloroplast, (b) mitochondria and (c) nuclear ribosomal RNA sequence of *M. jansenii*. (B) Dotplot of *M. jansenii* Hi‐C assembly against the (a) chloroplast, (b) mitochondria and (c) nuclear ribosomal RNA sequence of *M. jansenii*.
**Figure S2**. (a) Dotplots of Hi‐C pseudo‐molecules against HiFiasm contigs (longest contigs >1 Mb). (b) Dotplots of Hi‐C pseudo‐molecules against HiFiasm contigs (longest and middle size contigs).
**Figure S3**. (a) Dotplots of Hi‐C pseudo‐molecules against HiFiasm contigs (longest contigs >1 Mb). (b) Dotplots of Hi‐C pseudo‐molecules against HiFiasm contigs (longest and middle size contigs).
**Figure S4**. Chloroplast assembly covered by a single HiFiasm Contig (Ptg0000186|) and small bits by Ptg000066|.
**Figure S5**. Chloroplast sequence (Ptg0000186| and Ptg000066|) insertions in the Hi‐C assembly.
**Table S1**. IPA and HiFiasm assembly from different volumes of sequence data
**Table S2**. HiFiasm contigs (<1 Mb and >100 kb) that are part of Hi‐C pseudo‐molecule assembly
**Table S3**. HiFiasm contigs (biggest contigs and middle size contigs) corresponds to *Macadamia jansenii* Hi‐C 14 pseudo‐molecules

FigureS1‐S5

## Data Availability

The HiFiasm assembly, chloroplast assembly, mitochondria assembly and nuclear ribosomal RNA sequence of *M. jansenii* has been deposited under NCBI bioproject PRJNA694456.
