# Supplementary material for: De novo chromosome level assembly of a plant genome from long read sequence data
Source: Plant J. 2021 Dec 2;109(3):727–36. doi: 10.1111/tpj.15583 (PMC9300133; doi:10.1111/tpj.15583)
Supplement: Supplementary file 1 — Figure S1. (A) Dotplot of Macadamia jansenii Hifiasm longest contigs (more than 1 Mb) against the (a) chloroplast, (b) mitochondria and (c) nuclear ribosomal RNA sequence of M. jansenii. (B) Dotplot of M. jansenii Hi‐C assembly against the (a) chloroplast, (b) mitochondria and (c) nuclear ribosomal RNA sequence of M. jansenii. Figure S2. (a) Dotplots of Hi‐C pseudo‐molecules against HiFiasm contigs (longest contigs >1 Mb). (b) Dotplots of Hi‐C pseudo‐molecules against HiFiasm contigs (longest and middle size contigs). Figure S3. (a) Dotplots of Hi‐C pseudo‐molecules against HiFiasm contigs (longest contigs >1 Mb). (b) Dotplots of Hi‐C pseudo‐molecules against HiFiasm contigs (longest and middle size contigs). Figure S4. Chloroplast assembly covered by a single HiFiasm Contig (Ptg0000186|) and small bits by Ptg000066|. Figure S5. Chloroplast sequence (Ptg0000186| and Ptg000066|) insertions in the Hi‐C assembly. Table S1. IPA and HiFiasm assembly from different volumes of sequence data Table S2. HiFiasm contigs (<1 Mb and >100 kb) that are part of Hi‐C pseudo‐molecule assembly Table S3. HiFiasm contigs (biggest contigs and middle size contigs) corresponds to Macadamia jansenii Hi‐C 14 pseudo‐molecules [file TPJ-109-727-s002.docx]

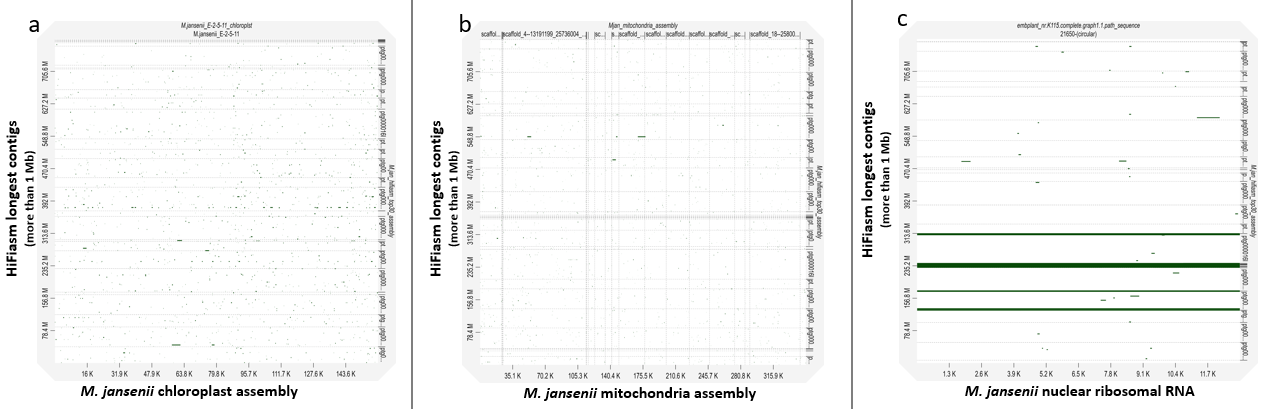


**Figure S1 (A):** Dotplot of *M. jansenii* HiFiasm longest contigs (more than 1 Mb) against a) Chloroplast, b) Mitochondria and c) Nuclear ribosomal RNA sequence of *M. jansenii*.


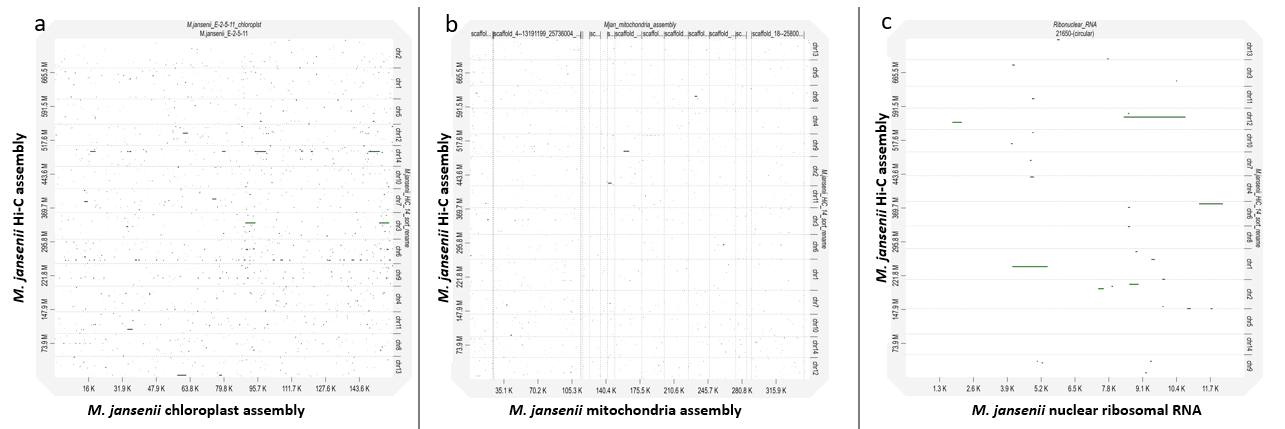


**Figure S1 (B)** Dotplot of *M. jansenii* Hi-C assembly against a) Chloroplast, b) Mitochondria and c) Nuclear ribosomal RNA sequence of *M. jansenii*.


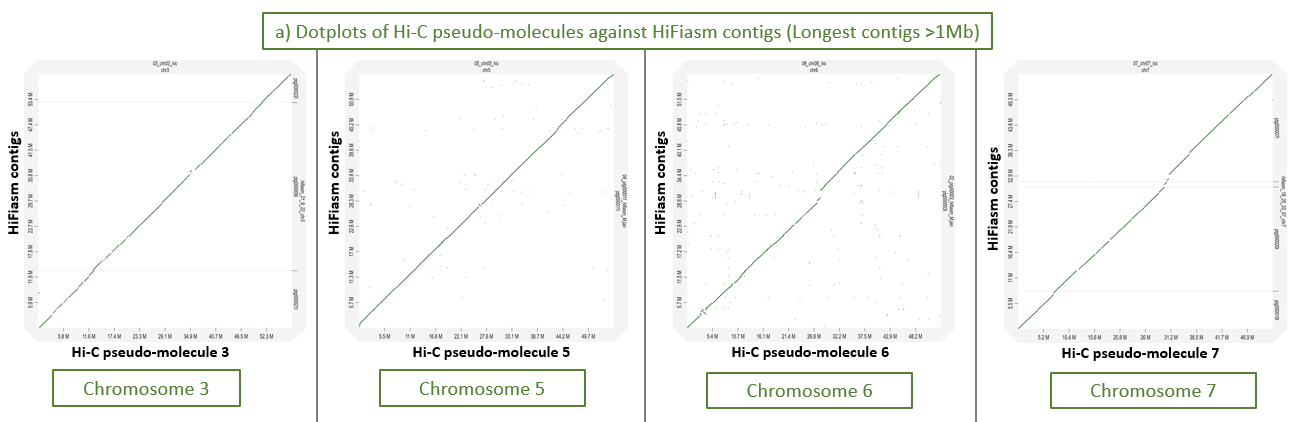


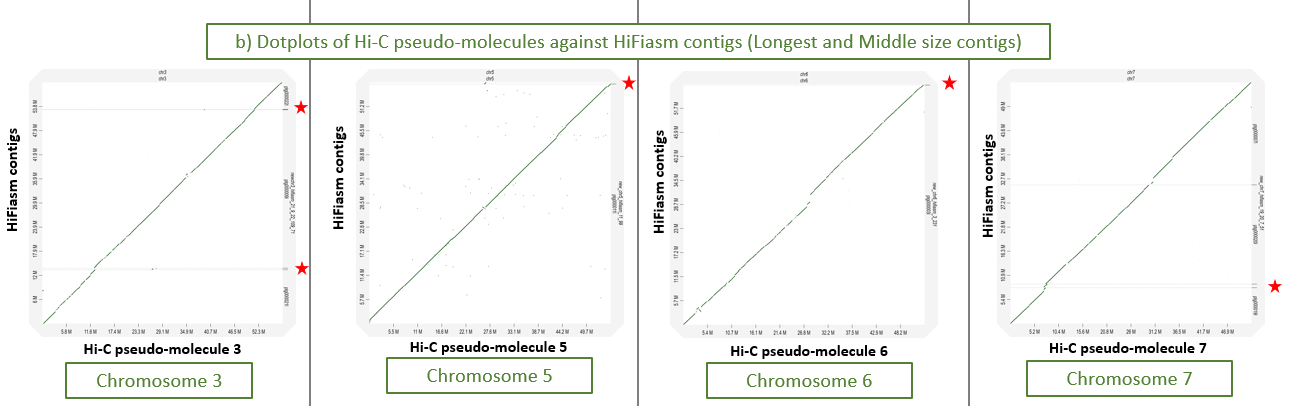


**Figure S2:** (a) Dotplots of Hi-C pseudo-molecules against HiFiasm contigs (Longest contigs >1Mb). (b) Dotplots of Hi-C pseudo-molecules against HiFiasm contigs (Longest and Middle size contigs). Red star showed the extra middle size contig (Table S3, 4^th^ column)


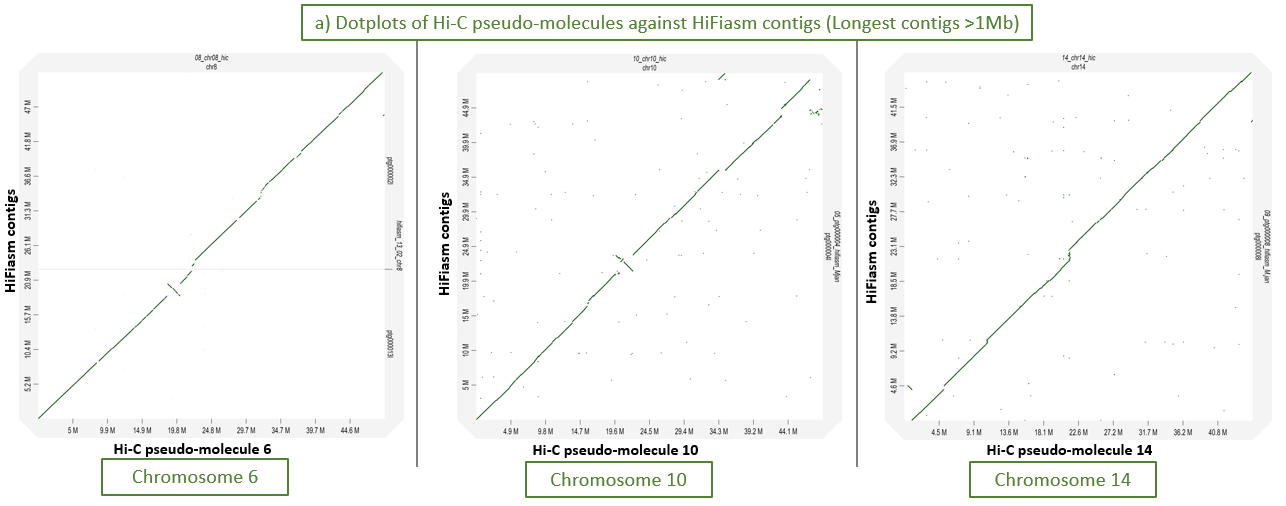


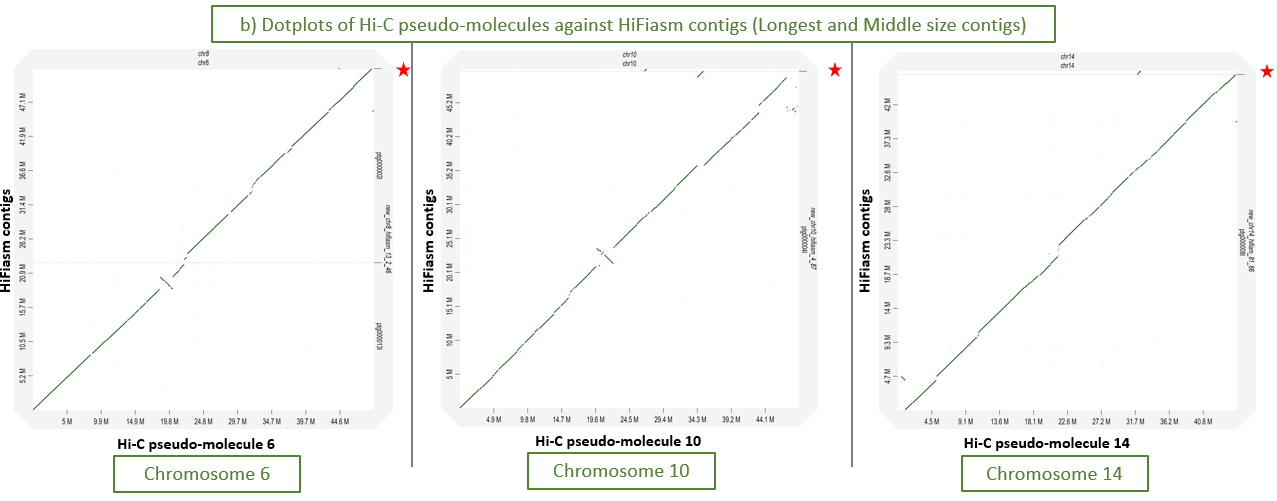


**Figure S3:** (a) Dotplots of Hi-C pseudo-molecules against HiFiasm contigs (Longest contigs >1Mb). S2 (b) Dotplots of Hi-C pseudo-molecules against HiFiasm contigs (Longest and Middle size contigs).


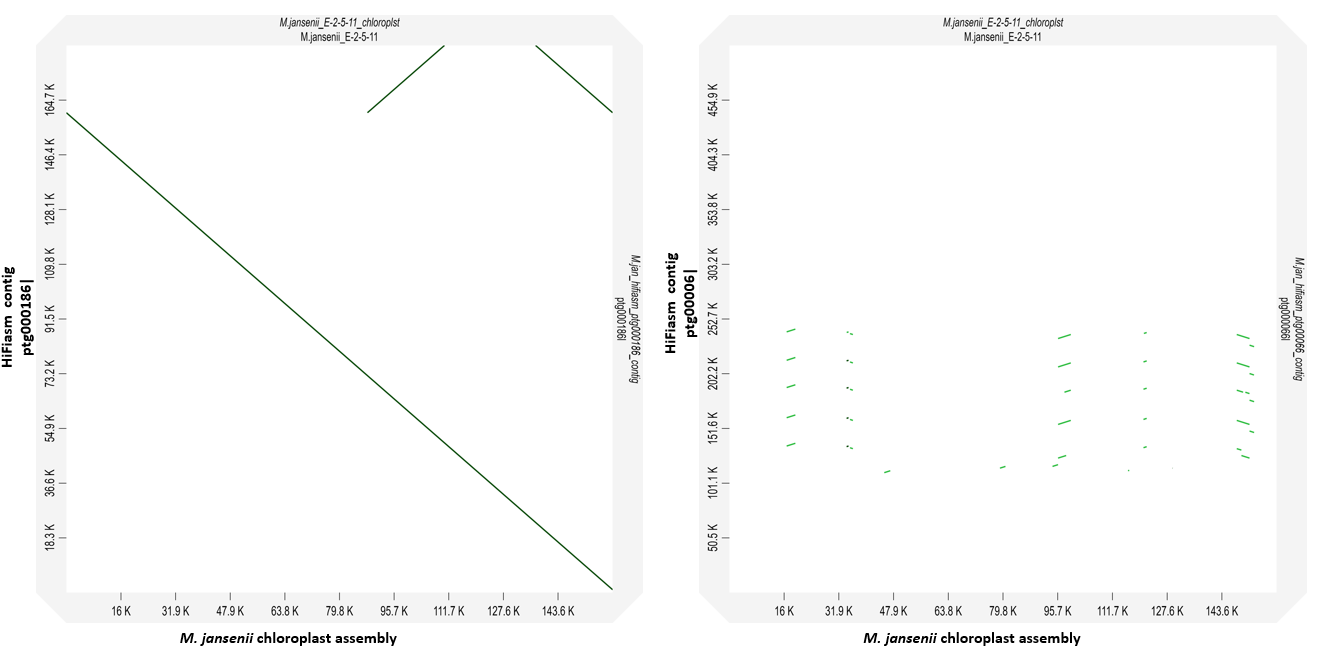


**Figure S4:** Chloroplast assembly covered by single HiFiasm Contig (Ptg0000186|) and small bits by Ptg000066|.


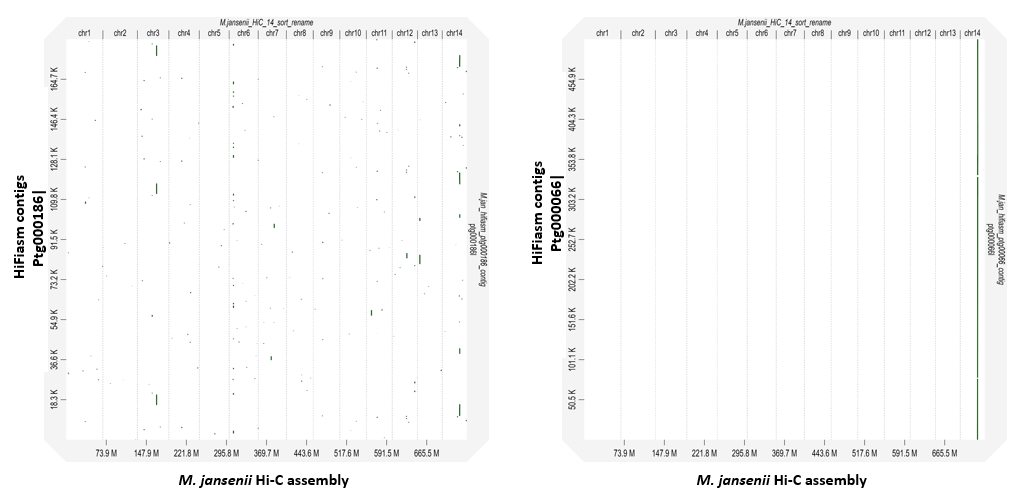


**Figure S5:** Chloroplast sequence (Ptg0000186| & Ptg000066|) insertions in the Hi-C assembly.

**Table S1:** IPA and HiFiasm assembly from different volumes of sequence data.

| **PacBio Sequence File details** | **Genome coverage** | **Assembler** | **Total number of contigs** | **Minimum length** | **Maximum length** | **Average length** | **N50** | **N75** | **Total length of assembly** | **BUSCO** |
| --- | --- | --- | --- | --- | --- | --- | --- | --- | --- | --- |
| CCS run 1 | 10 X | IPA | 1870 | 16 Kb | 3.5 Mb | 0.39 Mb | 0.61 Mb | 0.32 Mb | 736 Mb | 96.80% |
|  |  | HiFiasm | 4511 | 13 Kb | 7.2 Mb | 0.20 Mb | 0.38 Mb | 0.18 Mb | 909 Mb | 96.30% |
| CCS run 2 | 18 X | IPA | 728 | 13.7 Kb | 11.5 Mb | 1.00 Mb | 1.7 Mb | 0.99 Mb | 735 Mb | 95.70% |
|  |  | HiFiasm | 1058 | 15 Kb | 39.5 Mb | 0.79 Mb | 4.4 Mb | 2.1 Mb | 833 Mb | 98.40% |
| CCS run 1 + run 2 | 28 X | IPA | 284 | 11.4 Kb | 16.6 Mb | 2.6 Mb | 4.5 Mb | 2.6 Mb | 738 Mb | 98.00% |
|  |  | HiFiasm | 779 | 14.8 Kb | 71.9 Mb | 1 Mb | 46.1 Mb | 25.1 Mb | 826.7 Mb | 99.60% |

**Table S2:** HiFiasm contigs (< 1Mb and > 100 Kb) which are part of Hi-C pseudo-molecule assembly.

| **HiFiasm middle size contigs *** | **Length of contig** | **start** | **end** | **Strand** | **Hi-C chromosome** | **Length of chromosome** | **Start** | **end** | **Number of residue matches** | **Alignment block length** |
| --- | --- | --- | --- | --- | --- | --- | --- | --- | --- | --- |
| ptg000087l | 353246 | 6 | 333848 | + | chr10 | 48974653 | 26634412 | 26968299 | 319711 | 333951 |
| ptg000051l | 823842 | 510970 | 823838 | + | chr7 | 52077970 | 7635725 | 7942300 | 283252 | 314722 |
| ptg000109l | 388144 | 17 | 275074 | + | chr3 | 58143993 | 26511644 | 26786699 | 261393 | 275068 |
| ptg000099l | 388484 | 5047 | 272569 | + | chr5 | 55220784 | 26342363 | 26609914 | 259802 | 267725 |
| ptg000066l | 505394 | 92060 | 306715 | + | chr14 | 45288529 | 32032742 | 32247416 | 206578 | 214723 |
| ptg000071l | 117037 | 8 | 117031 | + | chr3 | 58143993 | 39146002 | 39262602 | 114711 | 117144 |
| ptg000231l | 173643 | 3 | 154150 | + | chr6 | 53595462 | 45346182 | 45497608 | 119329 | 166044 |
| ptg000046l | 103639 | 2 | 103632 | + | chr8 | 49563658 | 44424231 | 44527861 | 101007 | 103640 |

*All middle size contigs (#64) of HiFiasm assembly corresponds to Hi-C assembly, but only contigs with “Alignment block length” greater than 100 Kb were considered.

(These eight HiFiasm contigs which corresponds to seven pseudo- molecules of Hi-C, were then combined with original HiFiasm contigs (as shown in Table S3) and then dotploted against their corresponding Hi-C pseudo-molecule)

| ***M. jansenii* Hi-C pseudo-molecules** | **Size of Hi-C Pseudo-molecules** | **HiFiasm Contigs Corresponding to Hi-C scaffolds (> 1Mb)** | **HiFiasm Contigs Corresponding to Hi-C scaffolds (Middle_size contigs)** |
| --- | --- | --- | --- |
| Chr 1 | 67682215 | Ptg000016l (complete) | NA |
| Chr 2 | 63669590 | Ptg000006I (start small) + Ptg000025I + Ptg000010I (end large) | NA |
| Chr 3 | 58143993 | Ptg000021I (start small) + ptg000009l (middle large) + ptg000022I (end small) | ptg000109l + ptg000071l |
| Chr 4 | 56076407 | Ptg000017l (complete) | NA |
| Chr 5 | 55220784 | Ptg000011l (complete) | ptg000099l |
| Chr 6 | 53595462 | Ptg000003l (complete- fragmented) | ptg000231l |
| Chr 7 | 52077970 | Ptg000019I (start small) + Ptg000020I (middle) + ptg000033l + ptg000007I (last) | ptg000051l |
| Chr 8 | 49563658 | ptg000013I (start part) + ptg000002l (end part) | ptg000046l |
| Chr 9 | 49085581 | ptg000014l (start big) + ptg000005 (small end) | NA |
| Chr 10 | 48974653 | ptg000004l (complete but fragmented) | ptg000087l |
| Chr 11 | 47698009 | ptg000012l (complete) | NA |
| Chr 12 | 46713600 | ptg000001l (start half) + ptg000072I (middle tiny) + ptg000052I | NA |
| Chr 13 | 45610911 | ptg000023l (complete) | NA |
| Chr 14 | 45288529 | ptg000008l (complete) | ptg000066l |

**Table S3:** HiFiasm contigs (biggest contigs & middle size contigs) corresponds to *M. jansenii* Hi-C 14 pseudo-molecules.
