# Supplementary figures and images for: De novo chromosome level assembly of a plant genome from long read sequence data
Source: Plant J. 2021 Dec 2;109(3):727–36. doi: 10.1111/tpj.15583 (PMC9300133; doi:10.1111/tpj.15583)

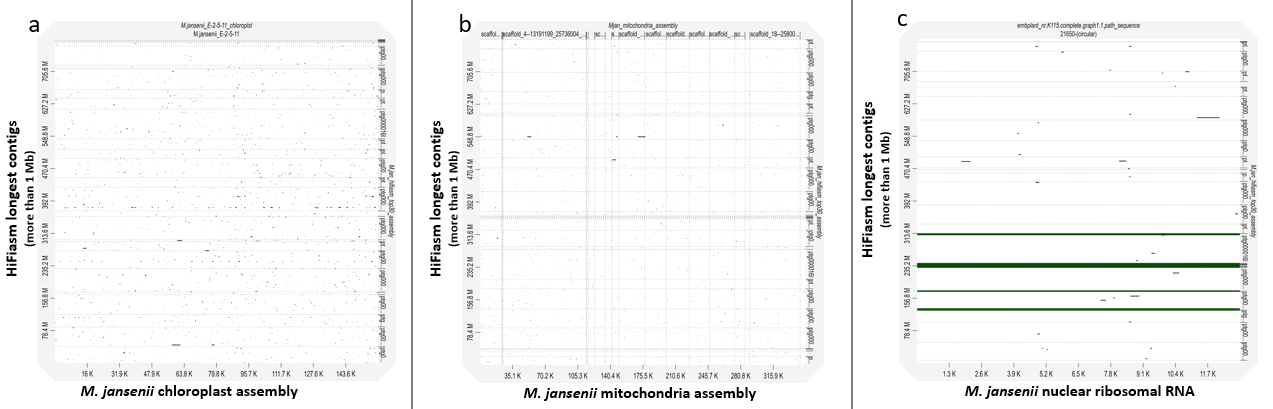

Supplement: Supplementary file 2 — FigureS1‐S5 [file TPJ-109-727-s001.zip › sup_fig_s1a.PNG]

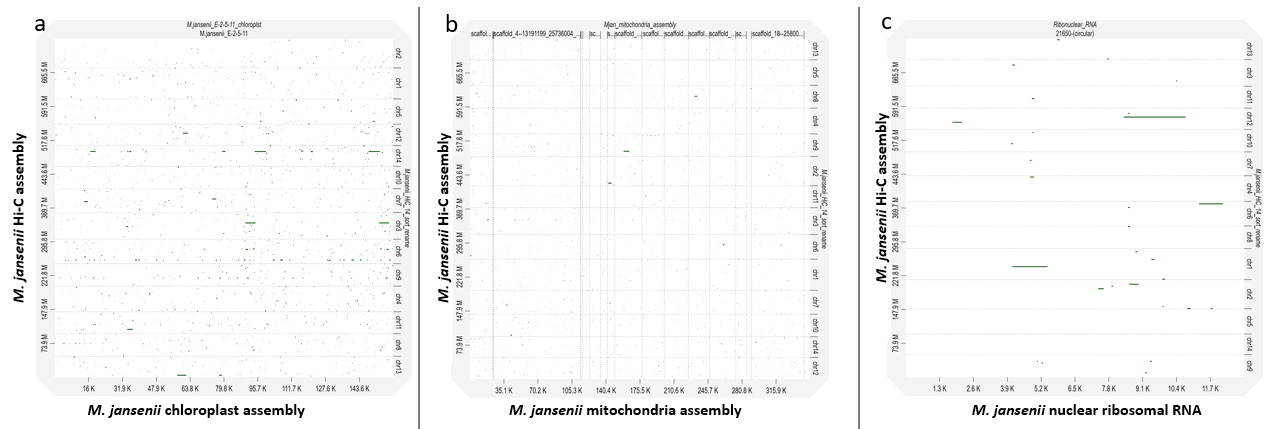

Supplement: Supplementary file 2 — FigureS1‐S5 [file TPJ-109-727-s001.zip › sup_fig_s1b.PNG]

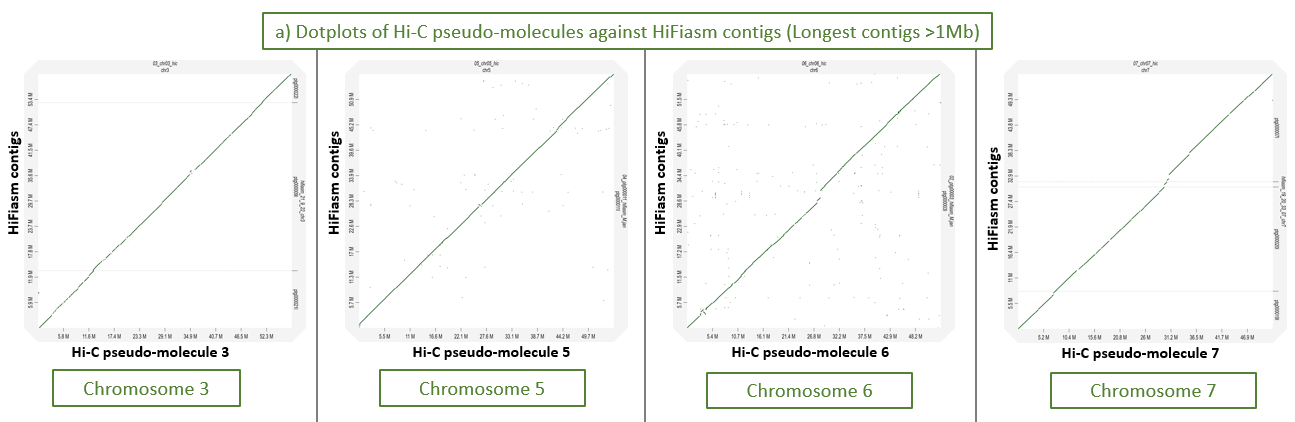

Supplement: Supplementary file 2 — FigureS1‐S5 [file TPJ-109-727-s001.zip › sup_fig_s2_1.PNG]

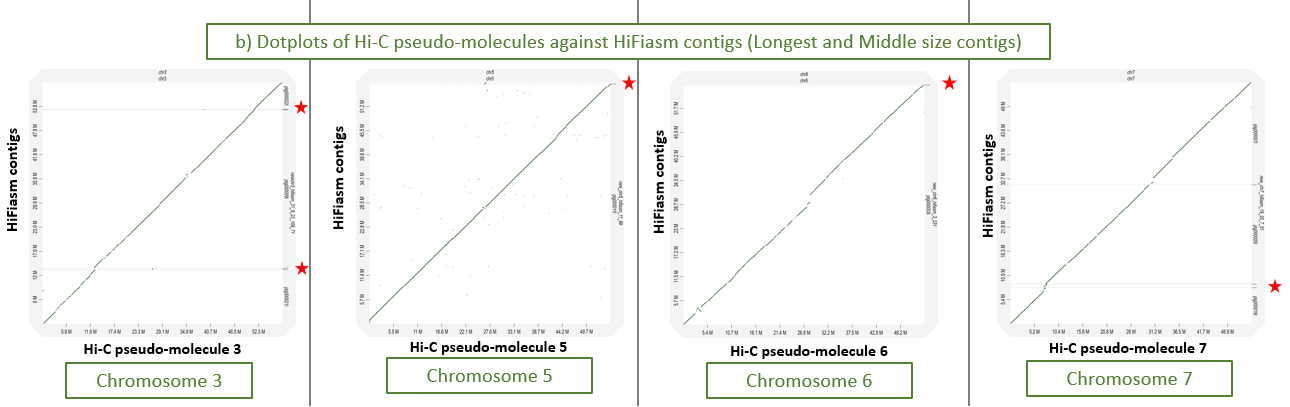

Supplement: Supplementary file 2 — FigureS1‐S5 [file TPJ-109-727-s001.zip › sup_fig_s2_2.PNG]

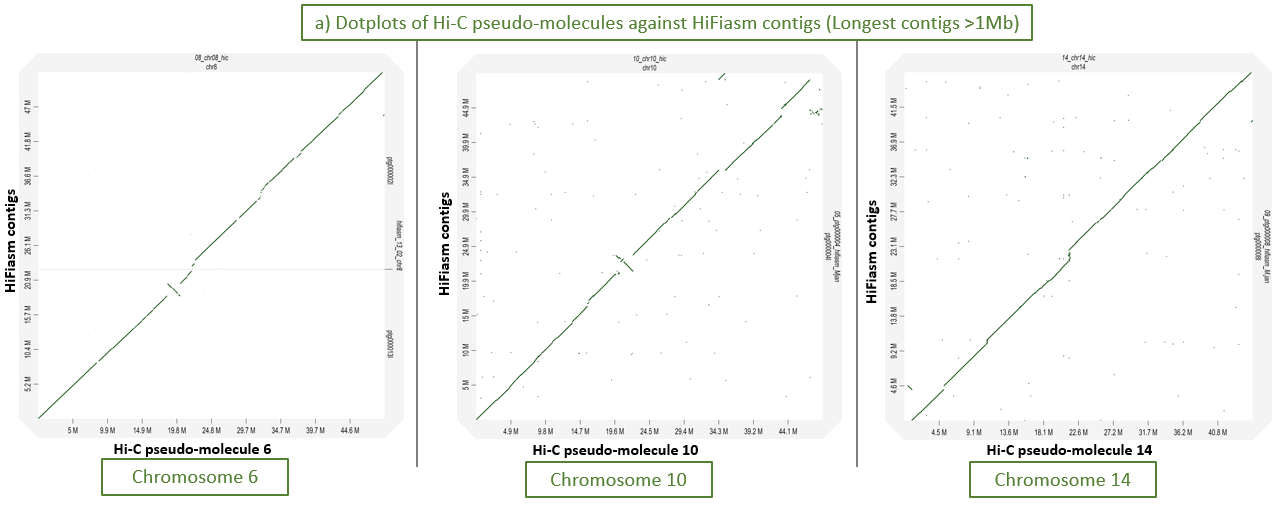

Supplement: Supplementary file 2 — FigureS1‐S5 [file TPJ-109-727-s001.zip › sup_fig_s3_1.PNG]

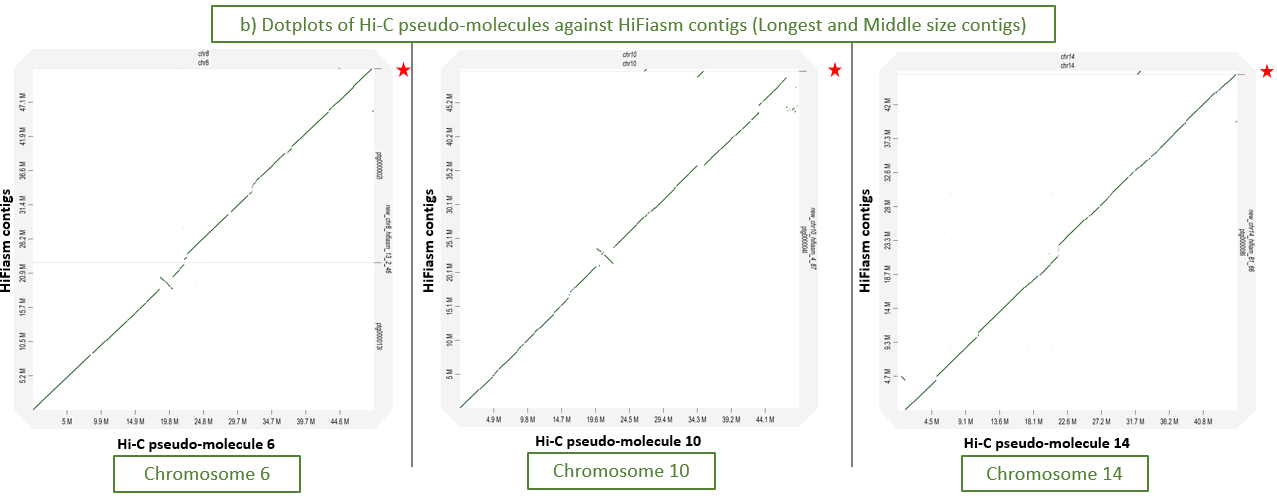

Supplement: Supplementary file 2 — FigureS1‐S5 [file TPJ-109-727-s001.zip › sup_fig_s3_2.PNG]

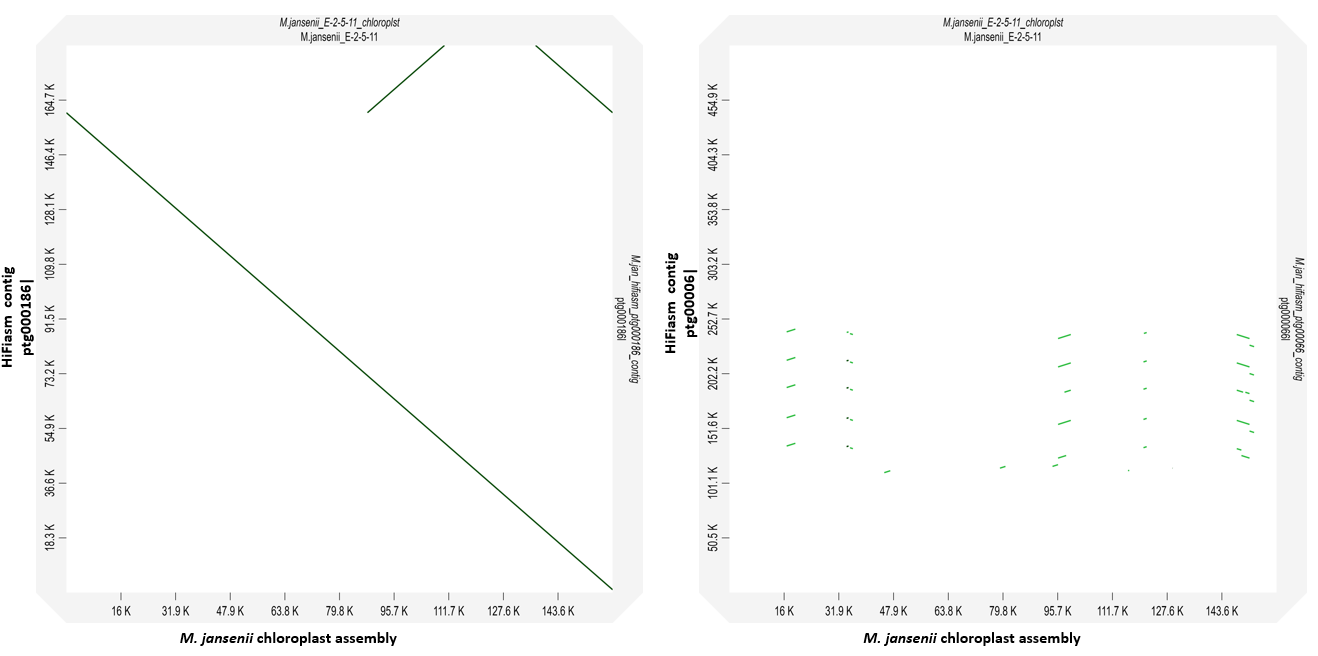

Supplement: Supplementary file 2 — FigureS1‐S5 [file TPJ-109-727-s001.zip › Sup_fig_s4.PNG]

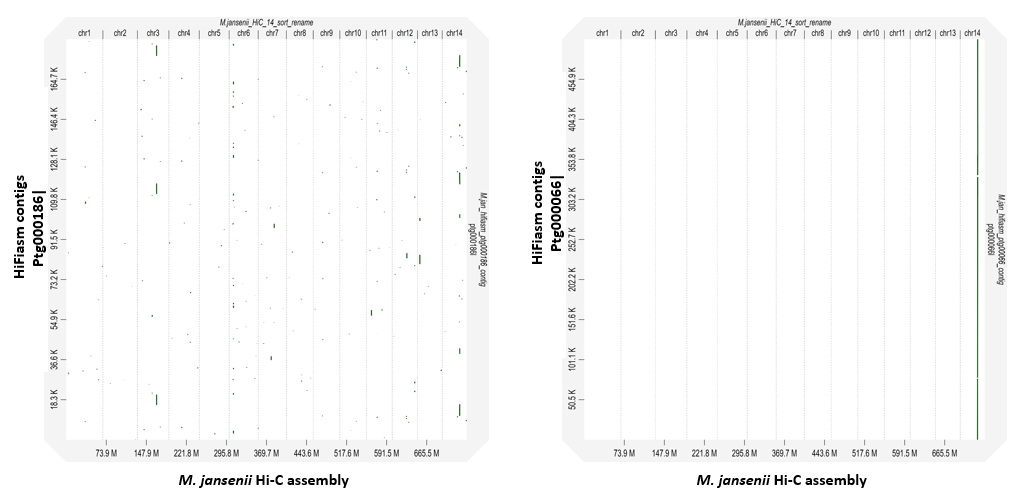

Supplement: Supplementary file 2 — FigureS1‐S5 [file TPJ-109-727-s001.zip › sup_fig_s5.PNG]
